# Supplementary material for: Rapid declines in systolic blood pressure are associated with an increase in pulse transit time
Source: PLoS One. 2020 Oct 8;15(10):e0240126. doi: 10.1371/journal.pone.0240126 (PMC7544103; doi:10.1371/journal.pone.0240126)
Supplement: Attachment — Submitted filename: Response to Reviewers.docx [file pone.0240126.s002.docx]

Dear reviewers,

Thank you very much for taking the time to review our submission. We very much appreciate your feedback. Below you will find our responses to your comments. Original comments are in blue italics, while our responses are in black standard text.

*Reviewer #1: In this paper, the authors examined the correlation between pulse transit time(PTT) and systolic blood pressure(SBP) from the waveform database of an intensive care unit to monitor the rapid declines in blood pressure. The results of this study can be expected to lead to non-invasive, real-time detection of rapid declines in blood pressure. The focus of the paper is well phrased, including the problems with the method. However, I would still like make the following suggestions to hopefully improve the quality of the paper:

1. (L 59-60) More properly, the vascular unloading technique was originally proposed by Marey, and later by Shirer and Penaz (Matsumura et al., 2017, IEEE Tran. Biomed. Eng., 64(5), 1131-1137). So, the author needs to revise the phrase “originally proposed by Penaz in 1973” with adding references. E. J. Marey, “Pression et vitesse du sang,” in Pbysiolotique Experimentale. vol. 2, Paris, France: G. Masson, 1876. Pp.307. H. W. Shirer, “Blood pressure measuring methods,” Ire Trans. Biomed. Electron., vol. BME-9, pp. 116–125, Apr. 1962. J. Penaz, “Photoelectric measurement of blood pressure, volume and flow in the finger,” in Proc. Dig. 10th Int. Conf. Med. Biological Eng., 1973, p. 104.*

Thank you for the correction; this has been updated in the manuscript.

*2. (L 78-79) A reference for conflicting papers should be added.*

The paragraph has been somewhat reformulated, and references have been added.

*3.(L 153-154) What was the reason for using 21 samples in the averaging of SBP, PTT, and ABP?*

An odd number of samples is required for Savitzky-Golay filtering. The choice to include 10 samples before and 10 after to determine the value at each heartbeat is somewhat arbitrary. At heart rates of 40-120 BPM, the length of 21 corresponds to an averaging over approximately 10-30 seconds. This was deemed to give a reasonable tradeoff, as a) beat-to-beat fluctuations which are clinically irrelevant are reasonably flattened and b) the lag time to detect BP-reductions is relatively short.

*4. (L 199-201) If the sample rate is 125 Hz, the time resolution should be 8 ms. However, why does Fig. 5(d) show a time resolution of 4 ms?*

This is an astute observation. Regrettably, a simple multiplication error was made when converting PTT from samples to milliseconds, multiplying by 4 instead of 8. As such, all PTT values reported in the manuscript must be multiplied by 2. This will be changed in the updated version of the manuscript. This unfortunate error does not affect the reported correlation coefficients, but will affect the estimate of ΔSBP/ΔPTT. Thank you for bringing this to our attention.

*5. (Supplementary analysis(3)) Did the authors include the values of SBP,PTT-RA,HR obtained by the missing/ambiguous peaks in the calculation of SBPAV,PTT_RAAV,HRAV? If it is not included, the analysis is the same as the main analysis, and I think that it is not a proof of robustness.*

For the supplementary analysis in question, the following method was applied: For ambiguous ABP-peaks, i.e. those that are deviant in shape or where multiple ABP-peaks appear between two ECG peaks, the tallest ABP-peak is included. Heartbeats with missing ABP-peaks, i.e. where there is no ABP-peak corresponding to the preceding ECG-peak, are indeed excluded as no measure of PTT can be obtained without the presence of both ECG and ABP-peaks. The exclusion of such heartbeats in real time should be a trivial task, achieved by a simple if-clause.

You are quite right, however, in insinuating that the presence of a large amount of missing peaks would limit the ability of the method, as there would be missing values for PTT. As such, we agree that the term robustness may be inaccurate in this instance. The main point that we wanted to make is that our results remain similar after including an additional 424 subjects, constituting an 83% increase in sample size. In retrospect, it may have been prudent to use more lax inclusion criteria for the main analysis. However, the criteria were predefined at 0 missing/ambiguous peaks in an attempt to isolate the physiological relationship of the variables. The challenge of obtaining high quality signals and reliable peak detection is clearly of paramount importance for real-life application, but it is not one that we claim to have solved in the current work. The statement claiming evidence of robustness (Revised manuscript L256-257) has been removed.

*Reviewer #2: The authors have investigated the relationship between declined systolic blood pressure (SBP) with pulse transit time (PTT) that was calculated from the ECG and arterial BP signals obtained in MIMIC database. The results indicate that the decrease of SBP is moderately associated with increase of PTT. Unlike the PTT calculated from ECG and photoplethysmogram (PTT), the PTT obtained from arterial BP would expect a higher correlation with BP, since it contains the direct information of BP rather than as an indirect estimate of BP. Therefore, I am not surprised with the results reported in this study. In addition, there are quite a lot study in the past decades or so on the relationship of the BP and PTT. I would therefore suggest the authors to clarify the significance of the study.*

While the ABP-signal contains direct information about SBP (peak height), we are not using the peak height in our analysis. We agree however that a somewhat higher correlation is expected, as the ABP-signal is obtained proximally to the major resistance vessels, in contrast to the PPG-signal. While many papers have examined the correlation between BP and PTT, we believe that our work includes some important factors:

i) We have specifically examined episodes of hypotension, which in our opinion represents the primary clinical point of interest in a theoretical cNIBP-system.
ii) We have provided clinical data on our patient population, enabling further discussion about applicability and confounding factors.
iii) We have examined within-subject data from more than one episode where available, enabling evaluation of the stability of the PTT-BP relationship over time.
iv) We have examined real-world data from hospitalized patients, likely covering a wide spectrum of pathologies and physiologic mechanisms for BP-reduction, as opposed to trials under experimental conditions.
v) Our sample size is quite large, exceeding most published work on the subject.

In our opinion, the novel aspects of our approach, as well as the combination of the aforementioned factors, constitute a valuable addition to the PTT/cNIBP literature. Two paragraphs have been added to clarify the novelties of the current investigation. (Revised manuscript L106-110 and L326-333)

*My other concerns are:*

1) PTT calculated from the peaks of arterial BP suffers from the reflective waveform which may impact the accuracy of the PTT;

It is true that the reflective nature of the arterial waveform may lead to difficulty in locating the exact position of the forward pressure wave peak. Indeed the forward wave and the reflecting wave will tend to conflate when measuring the pressure as peripherally as the radial artery, shifting the observed peak to the right. Under stable physiological conditions, the within-subject amount of shift may reasonably be assumed to be constant. In reality of course physiology is dynamic, and we are indeed looking at ‘unstable’ patients, progressing to hypotension. In cardiogenic and hypovolemic shock specifically, it has been described that reflected waves are separated more completely from the initial systolic peak [1]. The isolated effect of this phenomenon on our PTT metric would be a reduction in PTT during progression to shock. This effect would then oppose the inverse relationship between BP and PTT that we have found in our material. In summary, your concern about using the peaks of a waveform with a reflective component is absolutely valid. We initially considered using troughs or ΔBP_max_ instead of peaks for our analysis. We ultimately concluded that detection of peaks would be most reliable from a technical standpoint. Potential benefit from using e.g. troughs instead of peaks, specifically in subjects with cardiogenic/hypovolemic shock, would be of interest and may be a natural continuation of our work with the MIMIC database.

2) I understand the synchronization issue between ECG and PPT, what about the similar issue in ECG and arterial BP?

As mentioned in our manuscript, several researchers have noted that the sampling of the PPG signal is variable over time, which has been traced back to a Massimo module. To the best of our knowledge, no such issues have been described for the sampling of the ABP-waveform. In our current material, the sawtooth artifact that has been described in the PPG-derived PTT variable has not been observed in our ABP-derived PTT variable. There are still potential synchronicity issues, however. Firstly, there may be a constant offset between some ECG and ABP signals, which would lead to errors in the absolute values of PTT, but which should not affect the correlation between the two signals. Secondly, the sampling of the ECG signal was done with a variable offset, introducing random error in the PTT-metric. The presence of such random error may suggest that the correlation coefficients we present underestimate the correlation between variables.

3) For the moving average of PTT-RA, what is the time interval for the average?

See the answer to question 3 by reviewer 1.

4) In Fig. 6, there seems not too many data point for the estimate, but it was mentioned data from 511 subjects were included for the study

Fig. 6 pertains to supplementary analysis (2), comparing events for subjects who experienced more than one event. Only 215/511 subjects experienced more than one event that met inclusion criteria. Thus, the figure contains 215 data points, quite a few of which are clustered close to a bias of 0.

Kind regards,

Sebastian Grøvdal Schaanning & Nils Kristian Skjærvold
